# Phylogeography and Species Distribution Modeling of Guizhou Odorous Frog ( *Odorrana kweichowensis* ) Endemic to Southern China Karst Region

**DOI:** 10.1002/ece3.72160

**Published:** 2025-09-14

**Authors:** Li Shize, Liu Jing, Su Haijun, Wei Gang, Shen Tuo, Mu Lang, Xu Houqiang

**Affiliations:** ^1^ Laboratory of Animal Genetics, Breeding and Reproduction in the Plateau Mountainous Region, Ministry of Education, Collaborative Innovation Center for Mountain Ecology & Agro‐Bioengineering (CICMEAB), College of Life Sciences Guizhou University Guiyang Guizhou China; ^2^ College of Forestry Guizhou University Guiyang Guizhou China; ^3^ Biodiversity Conservation Key Laboratory Guiyang College Guiyang Guizhou China

**Keywords:** climate change, genetic diversity, karst region, suitable habitat

## Abstract

Karst in Southwest China, recognized as biodiversity hotspots, exhibits heightened sensitivity to climate change. The effects of climate change on these ecosystems via species distribution and population genetics remain inadequately investigated. This study presents the first genetic analysis of the endemic karst species *Odorrana kweichowensis* , utilizing mitochondrial gene fragments COI and ND2 to assess genetic structure and historical population dynamics. We further predict habitat suitability and genetic diversity under four climate scenarios for 2050 and 2070. Our findings reveal no significant genetic differentiation among populations, and the rapid expansion experienced in the past may have been driven by climate change. By 2050, except for the RCP8.5 scenario, we anticipate over 50% of haplotypes and sampling localities to persist. However, by 2070, only the RCP2.6 scenario maintains over 20% of haplotypes and nucleotide diversity, with the other scenarios predicting significant losses. Particularly under RCP8.5, all collected sampling localities face habitat loss. Nonetheless, suitable habitats persist near current protected areas. Thus, conservation efforts should prioritize the protection of habitats projected to remain beyond 2050, particularly those yet to be recognized as protected areas.

## Graphical abstract

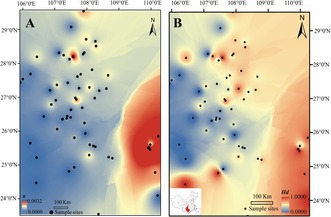

## 1 Introduction

Global climate change profoundly reshapes ecological and evolutionary processes across all levels of biological diversity (Carvalho et al. 2019; Rizvanovic et al. 2019). Manifested through shifts in temperature, precipitation, extreme weather events, and atmospheric/oceanic dynamics, these changes directly and indirectly intensify pressures on biodiversity (Parmesan 2006). Species respond by altering distributions (Lenoir et al. 2008; Crimmins et al. 2011; Fernández‐Cabello et al. 2025; Haase et al. 2025), phenologies (Vitasse et al. 2018), and physiological thresholds (Inouye 2009). However, when adaptations are insufficient, they can trigger ecosystem disruptions, including biological invasions and biodiversity loss (Hulme 2017; Mantyka‐Pringle et al. 2015).

The karst region of southwest China, a humid tropical–subtropical landscape shaped by geological and climatic forces (Wang et al. 2004, 2015; Yang et al. 2021), is characterized by rugged limestone topography, including towers, sinkholes, and subterranean drainage systems that create highly fragmented microhabitats with unique hydrological and microclimatic conditions. It stands as a globally significant biodiversity hotspot (Luken 1999; Myers et al. 2000), fostering endemism among taxa with specialized hydrological or microclimatic requirements (Wang et al. 2019; Yang et al. 2021). However, this environmental heterogeneity, rooted in unique geological structures, renders the region exceptionally vulnerable to climate change (Wang et al. 2019; Li, Liu, et al. 2021; Li, Wei, et al. 2021). Climate‐driven disruptions to the hydrological cycle exacerbate droughts, floods, rock desertification, and carbon cycle imbalances (Wang 2003; Lian et al. 2015; Peng et al. 2021), with altered precipitation (e.g., intensified drought‐flood cycles) and rising temperatures (e.g., enhanced evapotranspiration) amplifying karst‐specific risks like soil erosion and groundwater depletion. For instance, the endemic Chinese crocodile lizard ( *Shinisaurus crocodilurus* ) has already experienced habitat shrinkage due to such hydrological instability (Zhang et al. 2022).

Amphibians, with their permeable skin, aquatic larval stages, and limited dispersal, are hypersensitive to environmental changes (Carey and Alexander 2003; Li et al. 2013). Their strong site fidelity, strict habitat specificity, and temperature‐dependent development further heighten vulnerability to climate shifts (Li et al. 2013; McCallum 2010; Huang et al. 2017; Nneji et al. 2020; Zhao et al. 2020). Currently, 40.7% of amphibian species are globally threatened (Luedtike et al. 2023), with 43% of Chinese species significantly impacted (Jiang et al. 2016). Understanding their responses to climate change is thus critical for conservation.

The integration of ecological niche models (ENMs) and phylogeography provides a powerful framework for elucidating species' responses to environmental change, both past and future (Engler et al. 2017). ENMs utilize species occurrence data and environmental variables to probabilistically predict habitat suitability, enabling assessments of climate change impacts (e.g., Huang et al. 2017; Xu et al. 2015; Jin et al. 2016; Wu et al. 2018; Fan et al. 2019; Jiang et al. 2019, 2022). Phylogeography provides a complementary line of evidence by reconstructing historical population dynamics, which include dispersal, vicariance, and demographic shifts. These reconstructions reveal evolutionary responses to past environmental fluctuations and inform adaptive potential under future scenarios (Wang et al. 2017; Jiang et al. 2022). Together, these integrated approaches enable the quantification of how contemporary range contractions erode intraspecific genetic diversity. This erosion represents a critical loss that diminishes adaptive capacity and threatens long‐term survival (e.g., Pauls et al. 2013; Carvalho et al. 2019; Bálint et al. 2011; Rubidge et al. 2012; Jordan et al. 2016; Rizvanovic et al. 2019). However, despite the global recognition of karst ecosystems as biodiversity hotspots and climate change refugia, the relationship between range loss and genetic erosion varies substantially among species and regions (Karuno et al. 2023). This leaves a critical knowledge gap: The dynamics of range loss and genetic erosion remain poorly understood for taxa endemic to karst ecosystems, which are highly vulnerable to climate change due to their specialized habitat requirements.

The Guizhou Odorous Frog ( *Odorrana kweichowensis* ), recognized as a distinct species in 2018 (previously misidentified as *O. schmackeri* ; Li et al. 2018), is an ideal model to address this gap. Listed as Vulnerable (VU) on the IUCN Red List (IUCN SSC Amphibian Specialist Group 2020) due to habitat fragmentation and climate impacts, it is a karst obligate: Strictly dependent on streams and seepages within limestone formations, with physiological adaptations and limited dispersal binding it to hydrologically sensitive microhabitats (Li et al. 2018; Li, Liu, et al. 2021; Li, Wei, et al. 2021; Jiang et al. 2022). Notably, genetic analyses of 10 populations reveal a history of rapid expansion, suggesting a strong adaptive capacity to environmental fluctuations (Li, Liu, et al. 2021; Li, Wei, et al. 2021).

**FIGURE 1:**
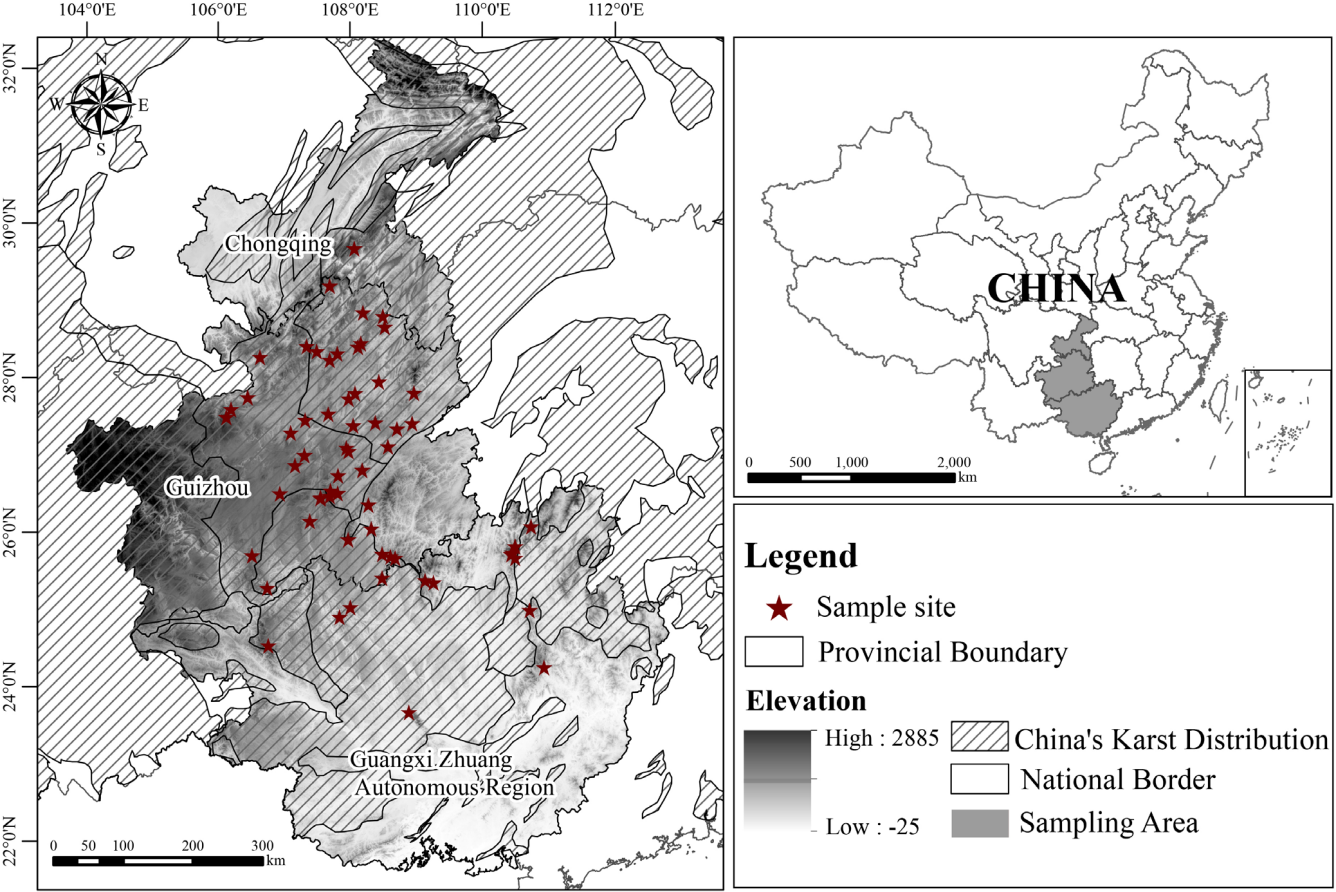
Sampling locality in this study.

Field surveys and literature records confirm *O. kweichowensis* endemism to karst regions of Chongqing, Guizhou, and Guangxi, China (Jiang et al. 2022; Li et al. 2015, 2018; Li, Liu, et al. 2021; Li, Wei, et al. 2021; Zhu 2016; Figure 1). In this study, we integrate phylogeography and ENMs to: (1) reconstruct *O. kweichowensis* ' population history and genetic structure, identifying drivers of its historical expansion; and (2) project how future climate change will impact its habitat suitability and genetic diversity. This work fills critical knowledge gaps in understanding climate change impacts on karst biodiversity, with implications for endemic species worldwide.

## 2 Materials and Methods

### 2.1 Sampling, DNA Extraction, and Sequencing

Based on previous studies (Jiang et al. 2022; Li et al. 2015, 2018; Li, Liu, et al. 2021; Li, Wei, et al. 2021; Zhu 2016), we conducted surveys in areas likely to be inhabited by *O. kweichowensis* . Noninvasive sampling was performed using buccal swab techniques: Individuals were gently captured, and a sterile cotton swab was rubbed against the inner cheek for 10 to 15 s to collect epithelial cells, and then released immediately at the capture site. All samples were immediately preserved in 99% anhydrous ethanol. A total of 716 individual samples from 62 localities were collected (Figure 1, Table S1). The Animal Care and Use Committee of Guizhou University provided full approval for this research (Number: EAE‐GZU‐2022‐T115), ensuring compliance with ethical guidelines for animal handling.

Genomic DNA was extracted from each sample using a Tissue Kit (Qiagen). Samples were subjected to PCR amplification and sequencing for two mitochondrial genes: cytochrome oxidase subunit I (COI) and NADH dehydrogenase subunit 2 (ND2). COI amplification utilized primers Chmf4 (5′‐TYTCWACWAAYCAYAAAGAYATCGG‐3′) and Chmr4 (5′‐ACYTCRGGRTGRCCRAARAATCA‐3′) following Che et al. (2012), while ND2 used Ile‐LND2 (5′‐ATAGGGAGACTTATAGGGGTTC‐3′) and Asn‐HDN2 (5′‐CTAAGTCATTACGGGATCGAGGCC‐3′) following Li et al. (2015). PCR amplification reactions were performed in a 30 μL volume containing: 1× High‐Fidelity Master Mix (Chengdu TSINGKE Biological Technology Co. Ltd.) 15 μL, ddH_2_O 10 μL, 0.5 μM forward primer 2 μL, 0.5 μM reverse primer 2 μL, and DNA template (4.25 μg/μL) 1 μL. The reaction conditions were as follows: an initial denaturing step at 95°C for 4 min; 36 cycles of denaturing at 95°C for 40s, annealing at 46°C (for COI)/52°C (for ND2) for 40 s, and extending at 72°C for 70 s, and a final extending step of 72°C for 10 min. PCR products were purified with spin columns and then sequenced in both directions using the same primers as for the PCR amplification. Sequencing was performed on an ABI Prism 3730 automated DNA sequencer at Chengdu TSINGKE Biological Technology Co. Ltd. (Chengdu, China). All sequences have been deposited in GenBank under accession numbers PP812697‐PP813412 for COI and PP813876‐PP814591 for ND2 (Table S1).

### 2.2 Phylogenetic Analyses

Sequences were assembled and aligned using the ClustalW module in BioEdit 7.0.9.0 with default settings (Hall 1999). Phylogenetic trees were reconstructed from the concatenated COI and ND2 dataset using maximum likelihood (ML) in PhyML 3.0 (Guindon et al. 2010) and Bayesian Inference (BI) in MrBayes 3.12 (Ronquist and Huelsenbeck 2003). The optimal partitioning scheme and evolutionary model for each partition were selected using PartitionFinder v2.1.1 (Lanfear et al. 2016), applying the Bayesian information criterion (BIC) to define partitions by codon position. For ML analysis, branch support was assessed using 10,000 nonparametric bootstrap replicates. For BI analysis, two independent runs of four Markov chains each were performed for 60 million generations, sampling every 1000 generations. The first 25% of generations were discarded as burn‐in; Bayesian posterior probabilities were then calculated, and a majority‐rule consensus tree was constructed from the remaining samples.

### 2.3 Population Structure and Dynamics History

Haplotype diversity (*Hd*) and nucleotide diversity (*π*) were calculated from mitochondrial data using DnaSP v5.1 (Librado and Rozas 2009). Neutrality tests, including Tajima's D and Fu's FS (Tajima 1989; Fu 1997), as well as mismatch distribution analyses assessing the sum of squared differences (SSD) and Harpending's raggedness index (*Hrag*) were conducted based on mitochondrial DNA (mtDNA) for all sampling localities using Arlequin v3.0 (Excoffier et al. 2005). The mismatch distribution curve was plotted using DnaSP v5.1. Bayesian skyline plots (BSP) were generated using BEAST 1.8.4 to infer population history and effective population size changes over time (Drummond et al. 2005, 2012). Calibration of the molecular clock was based on a substitution rate of 0.65% per million years for mtDNA, a rate commonly used for amphibian ND1 and COI genes (Macey et al. 1998, 2001; Wang et al. 2017). The analysis was conducted under a strict molecular clock model, a GTR site model, and a Coalescent Bayesian skyline prior. BEAST runs were performed twice for 1 × 10^9^ generations, with sampling every 10,000 generations, and each run was assessed to ensure the posterior probability distribution had an ESS > 200 for all parameters after a 10% burn‐in. Haplotype files were generated using DnaSP v5.1, and haplotype networks were constructed using the TCS network module in PopART v1.7 (Clement et al. 2000; Leigh and Bryant 2015). To assess genetic structure, hierarchical analysis of molecular variance (AMOVA) was performed using ARLEQUIN v3.5.1 (Excoffier et al. 2005) to partition genetic variation among sampling localities, among groups of sampling localities (if defined), and within sampling localities. Pairwise Fst values between sampling localities were calculated in ARLEQUIN v3.5.1 to estimate genetic distances and were tested for significance using 100,000 permutations. Geographic distances between sampling localities were computed as Euclidean distances using the Geographic Distance Matrix Generator v1.2.3 (Ersts 2012) based on GPS coordinates (decimal degrees). Mantel tests with 10,000 randomizations were performed in the “vegan” package in R to assess the correlation between pairwise genetic distances (Fst/(1‐Fst)) and pairwise geographic distances, testing the isolation‐by‐distance (IBD) model.

### 2.4 Species Distribution Model

We compiled 170 occurrence records from fieldwork and literature (Table S2). To reduce sampling bias, records located < 1 km apart were thinned using the “spThin” R package (Aiello‐Lammens et al. 2015), resulting in 165 records.

We obtained 19 bioclimatic variables and elevation (Alt) for the 1970–2000 baseline period from WorldClim 2.1 (30 arc‐second resolution; Fick and Hijmans 2017; Table S4). For paleoclimatic periods (the last interglacial [LIG: ~120,000–140,000 years ago], the last glacial maximum [LGM: ~22,000 years ago], and the mid‐Holocene [MID: ~6000 years ago]), we used bioclimatic variables resampled to 30 arc‐second resolution from WorldClim 1.4 data (Hijmans et al. 2005). Elevation was held constant across all time periods following recommendations for paleodistribution modeling (Svenning et al. 2011). Elevation was retained in the models because: (1) it directly influences microhabitat suitability via temperature lapse rates (0.6°C/100 m) and hydrology (Dobrowski 2011); (2) the species' distribution is strongly linked to karst topography (Figure 1); and (3) precedent exists for including elevation in amphibian ENMs (e.g., Jiang et al. 2022; Li, Liu, et al. 2021; Li, Wei, et al. 2021).

For future projections (2041–2060 [2050s]; 2061–2080 [2070s]), we used four representative concentration pathways (RCPs), greenhouse gas emission scenarios representing different climate futures based on radiative forcing levels (in W/m^2^): RCP2.6 (low emissions), RCP4.5 (intermediate stabilization), RCP6.0 (intermediate‐high), and RCP8.5 (high emissions) (Fick and Hijmans 2017).

To mitigate potential model overfitting from collinear environmental variables (Hu et al. 2016), we assessed pairwise correlations among the 20 variables (Table S3). Following Dormann et al. (2013), who recommend *r* > 0.7 as a conservative threshold to avoid multicollinearity effects that can distort parameter estimates and inflate model variance, we excluded variables with Pearson's *r* > 0.8 to ensure robust variable selection. This resulted in the selection of eight variables: Bio02 (Mean diurnal range), Bio03 (Isothermality), Bio08 (Mean temperature of wettest quarter), Bio09 (Mean temperature of driest quarter), Bio12 (Annual precipitation), Bio17 (Precipitation of driest quarter), Bio18 (Precipitation of warmest quarter), and Alt (Elevation) for use in model construction (Figure S1).

ENMs were developed and optimized using the “Kuenm” package (Cobos et al. 2019). Model performance was evaluated using the AUC statistic (Swets 1988): poor (0–0.6), fair (0.6–0.7), good (0.7–0.8), very good (0.8–0.9), excellent (0.9–1). Habitat suitability rasters were classified into categories in ArcGIS 10.4 (Esri 2016) using the maximum training sensitivity plus specificity (MTSS) threshold: unsuitable (< MTSS), low (MTSS—0.5), moderate (0.5–0.7), high (> 0.7).

### 2.5 Quantifying Erosion of Genetic Diversity by Climate Change

To assess the erosion of genetic diversity caused by projected climate change (following Karuno et al. 2023), we (1) associated mtDNA sequences with collection localities; (2) identified sequences originating from localities projected to lose suitable habitat under future climates; (3) computed the losses in the number of unique haplotypes and in nucleotide diversity relative to current conditions; (4) calculated the proportion of unique haplotypes and of π lost. Habitat suitability changes between current and future periods were estimated using ENMs Toolbox v2.4 (“Distribution changes between binary”). Raster files were visualized in ArcMap 10.4.

## 3 Results

### 3.1 Phylogenetics Based on mtDNA

The concatenated COI + ND2 alignment comprised 1461 bp (COI: 579 bp; ND2: 882 bp). No indels were detected, and all sequences translated accurately into amino acids using vertebrate mitochondrial codes. Among 716 *O. kweichowensis* individuals from 62 localities, we identified 94 unique haplotypes.

**FIGURE 2:**
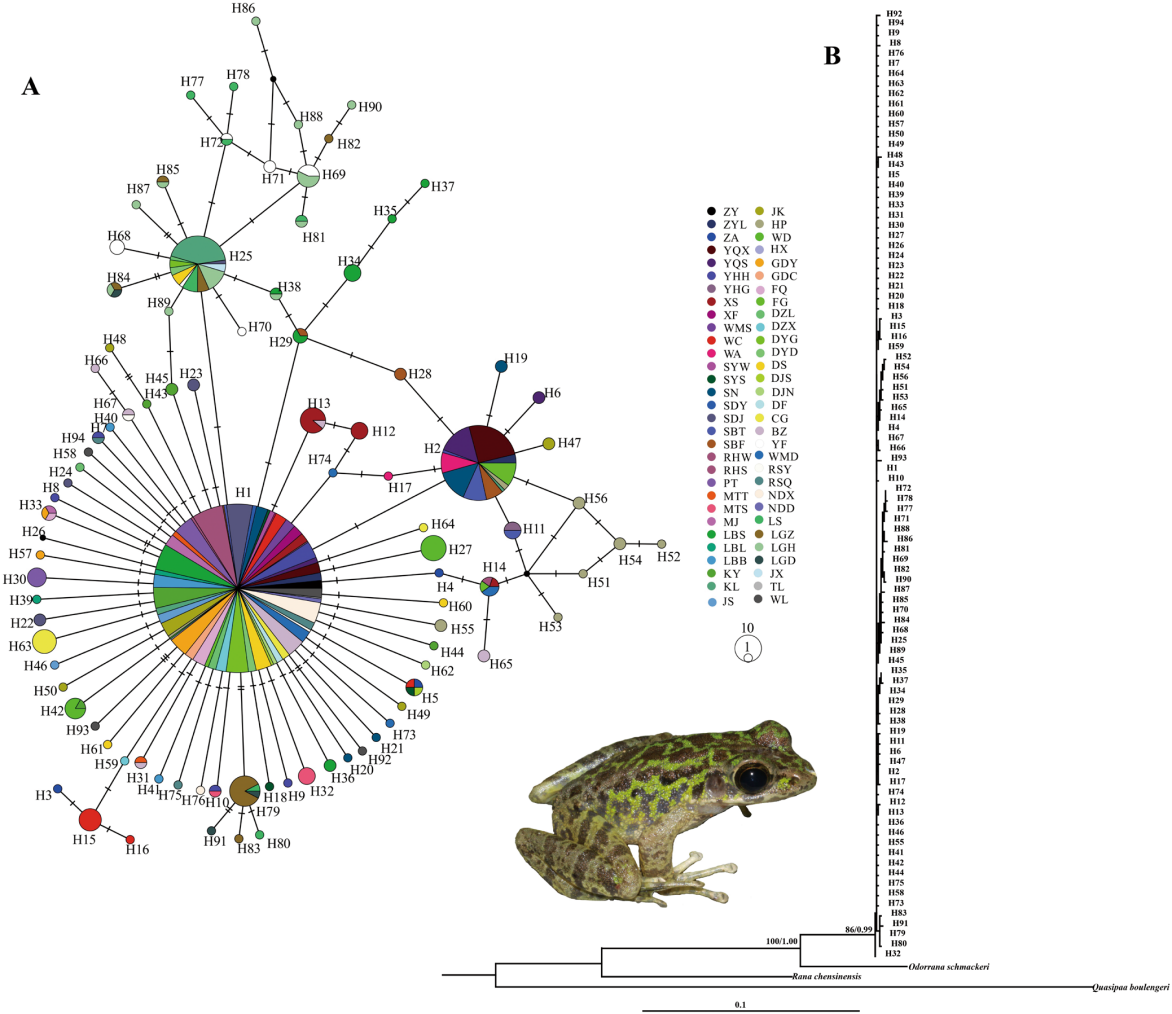
Haplotype network and ML tree of the *O. kweichowensis* based on the COI and ND2 genes. (A) Haplotype network. Each circle represents a unique haplotype, (denoted as “H” followed by a number, e.g., H1), with circle size proportional to haplotype frequency. Colors correspond to geographic regions (see legend for locality codes). (B) Maximum likelihood (ML) tree based on concatenated COI and ND2 sequences, with node support values (bootstrap/posterior probability) indicated.

PartitionFinder v2.1.1 selected the optimal partitioning scheme under BIC: two partitions by gene (COI: TN93 + G; ND2: GTR + I + G). Both ML and BI analyses produced congruent topologies (Figure 2B), revealing no significant divergence among haplotypes. The star‐shaped haplotype network further supports limited phylogenetic structure (Figure 2A).

### 3.2 Population Genetic Structure

Haplotype distribution analysis revealed pronounced spatial heterogeneity: H1 was the dominant haplotype, occurring in 398 individuals (55.6% of all samples) across 54 sampling localities, indicating panmictic gene flow; H2 showed regional concentration, appearing in 79 individuals from 10 localities exclusively in northeastern Guizhou; H25 clustered in 44 individuals from 10 localities within the Guangxi–Guangzhou karst corridor and the KL uniquely harbored both widespread (H1) and regional haplotypes (H2, H25), suggesting a contact zone between dispersal lineages (Table S4).

**FIGURE 3:**
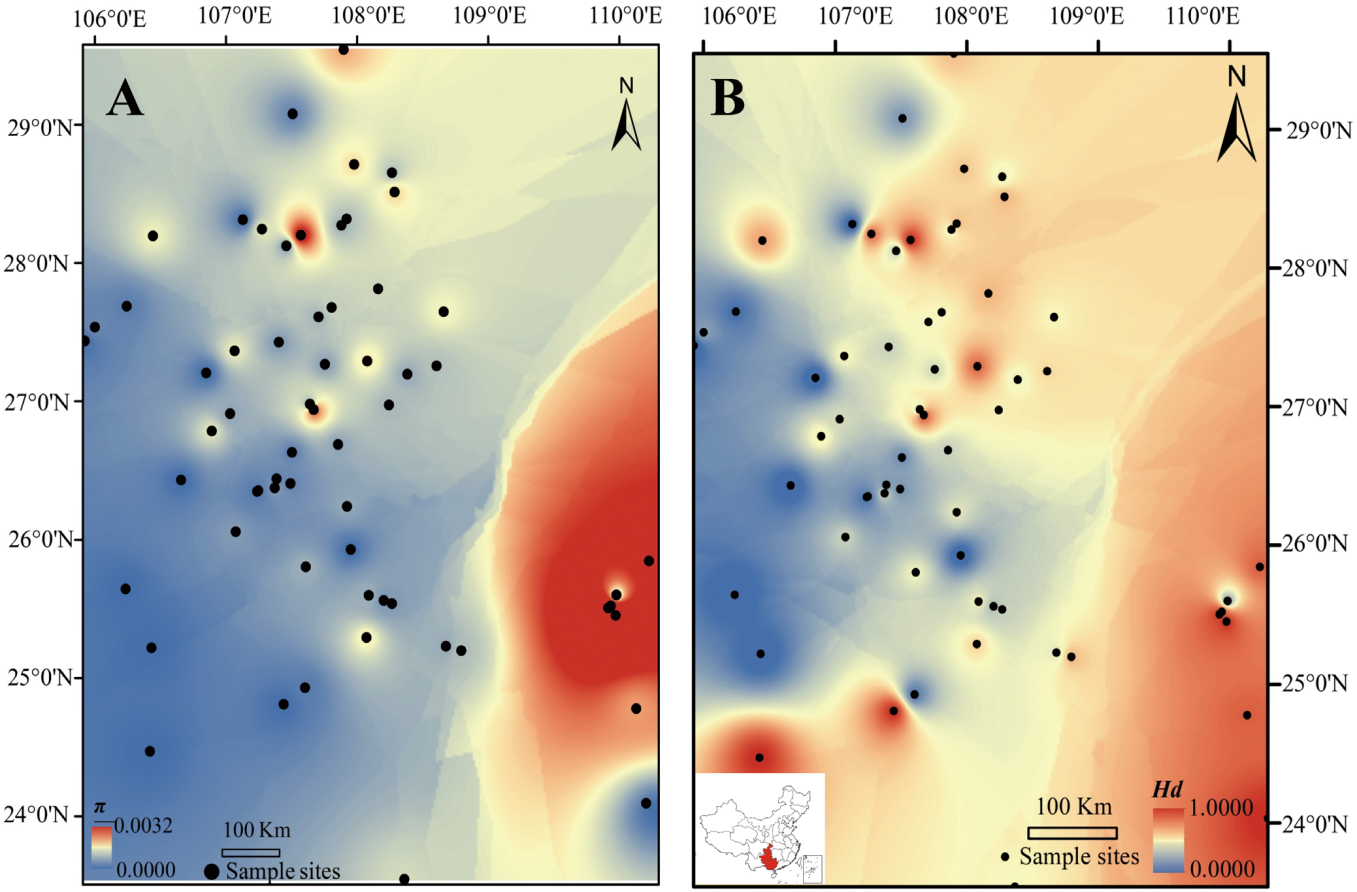
Spatial patterns of genetic diversity in *O. kweichowensis* . (A) Nucleotide diversity (*π*). (B) Haplotype diversity (Hd). Warmer colors indicate higher genetic diversity. Black dots represent sampling localities. The inset map in the bottom‐left corner shows the location of the study area (highlighted in red) within China.

Genetic diversity varied markedly across localities: *Hd* ranged from 0 to 1.00 (0.67 ± 0.02) and *π* from 0 to 0.00319 (0.00082 ± 0.0001), with the highest diversity clustered in southwestern localities (Figure 3). AMOVA indicated significant but weak subdivision (*p* < 0.001): 38.0% variance among localities (pairwise Fst range: 0.000–0.778), 42.2% within localities (Table S5). The nonsignificant isolation‐by‐distance pattern (Mantel test: *R* = 0.1302, *p* = 0.101, Figure S2) implies stochastic divergence in karst microhabitats rather than distance‐driven isolation.

### 3.3 Population Demographic History

**FIGURE 4:**
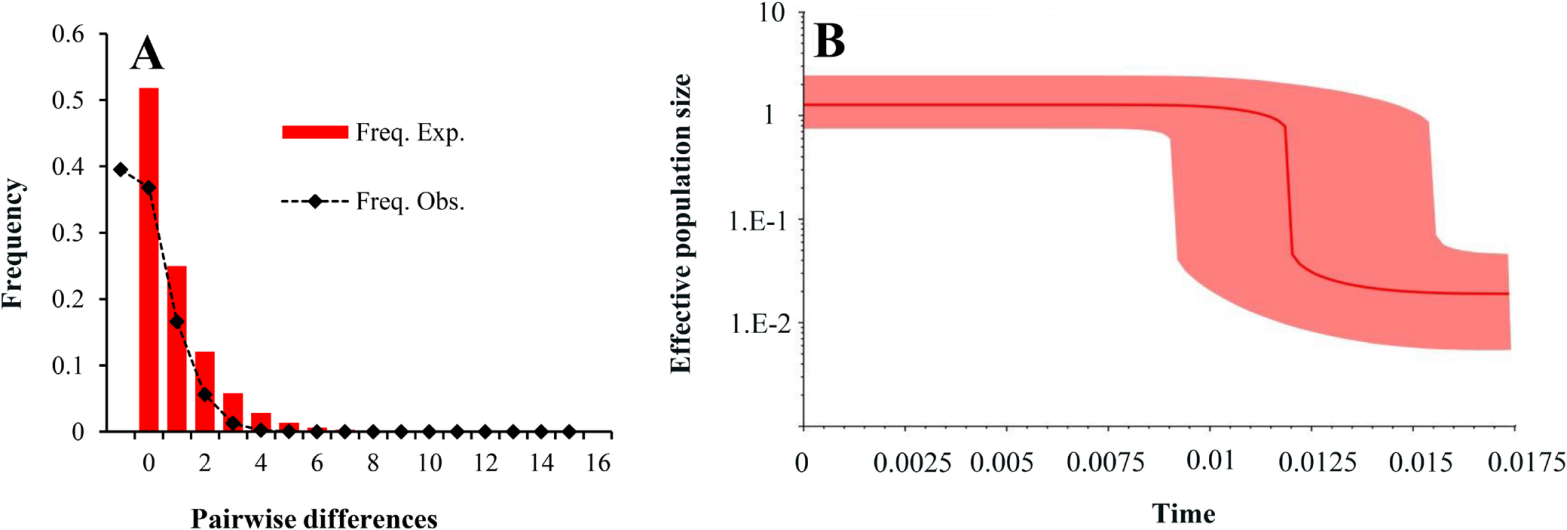
Mismatch distributions (A) and Bayesian skyline plot (B) estimated by BEAST of *O. kweichowensis* . In the Bayesian skyline plot, *x*‐axis, time in millions of years (Ma); *y*‐axis, effective population size (the product of effective population size and generation length in Ma), mean estimate and both 95% HPD limits are indicated.

From the neutrality tests and mismatch distribution analysis, we observed consistent patterns that suggest *O. kweichowensis* has recently experienced population growth. Specifically, the significantly negative values obtained from Tajima's *D* test (−2.30789, *p* = 0.000 < 0.001) and Fu's FS test (−28.12721, *p* = 0.000 < 0.001) serve as evidence for the recent expansion. Additionally, the nonsignificant results of the *SSD* are 0.00003 (*p* = 0.890 > 0.05) and *HRI* are 0.055726 (*p* = 0.540 > 0.05) indicate that there is no significant deviation between the observed mismatch distribution and the simulation under a model of sudden population growth. This finding is further supported by the mismatch distribution analysis and the BSP. The unimodal distribution drawn from the overall population in the mismatch distribution analysis (Figure 4A) suggests a trend of population growth, and in the BSP, the expansion event is inferred to have occurred approximately 15,000 years ago (Figure 4B).

### 3.4 Species Distribution Modeling and Ancestral Locations

The most suitable model for *O. kweichowensis* was identified as quadratic and product (pq) with a regularization multiplier of 1.9. The resulting AUC of 0.902 (Figure S3) demonstrates the excellent overall predictive ability of our ENMs, and the MTSS was 0.3893.

The MaxEnt model identified three critical factors limiting *O. kweichowensis* distribution: Bio9 (33.8% contribution) with a narrow adaptation range of −2.5°C to 6.6°C, beyond which suitability sharply declines; Bio17 (26.7% contribution) requiring 74.1–148.1 mm to maintain viable sampling localities, as lower values jeopardize karst aquifer recharge; and Alt (15.8% contribution) confining the species to 10.5 to 1040 m where microclimates buffer climatic extremes (Figure S4). This triple constraint (−2.5°C ≤ Bio9 ≤ 6.6°C; 74.1 mm ≤ Bio17 ≤ 148.1 mm; Alt ≤ 1040 m) defines the species' dry‐season climate refuge, with future habitat loss under high‐emission scenarios (e.g., RCP8.5) directly linked to Bio9 exceeding 6.6°C and/or Bio17 falling below 74.1 mm.

In the last interglacial period, no suitable habitats were predicted. However, during the LGM, suitable areas were identified in the southeastern region of Guizhou, at the tri‐junction of Guizhou, Chongqing, and Hunan, the northwestern part of Hubei, and the northeastern part of Yunnan, respectively, located in the Miaoling Mountains, the Wuling Mountains, and the Wumeng Mountains, which may imply that these areas were refuges during the LIG. In the MID, the spatial distribution of suitable areas remained largely unchanged, although the extent of these habitats increased significantly. In the present, the range of suitable habitats has expanded markedly, extending from the junction of Hubei and Chongqing to the junction of Yunnan and Sichuan, and to the intersection of Guizhou and Guangxi. Additionally, suitable habitats have also been detected in the northeastern part of Guangxi, as well as in the border areas between eastern Guangxi and western Guangdong. Highly suitable habitats are predominantly located in the eastern region of Guizhou and in certain central and northern areas (Figure S5).

### 3.5 Suitable Range Size Changes Under the Future Climate Scenarios

**FIGURE 5:**
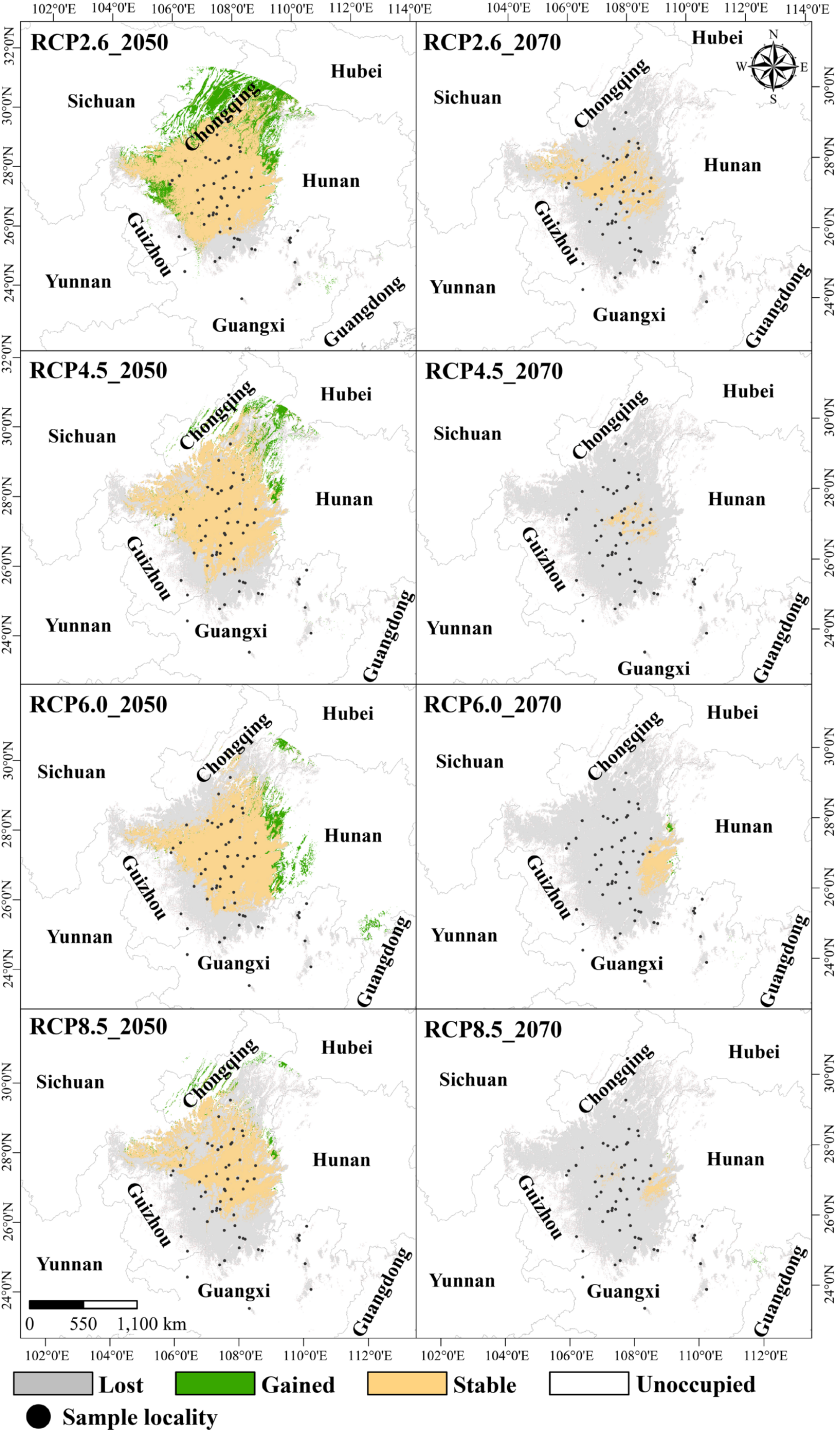
Suitable range size changes under the future climate scenarios of *O. kweichowensis* . Colors indicate habitat status: Gray = contraction (loss of current suitable habitat), green = expansion (new suitable habitat), orange = stable (persistent suitable habitat). RCPs (Representative Concentration Pathways) are greenhouse gas emission scenarios: RCP2.6 (low emissions), RCP4.5 (intermediate‐stabilization), RCP6.0 (intermediate‐high), and RCP8.5 (high emissions), representing increasing levels of future warming.

**FIGURE 6:**
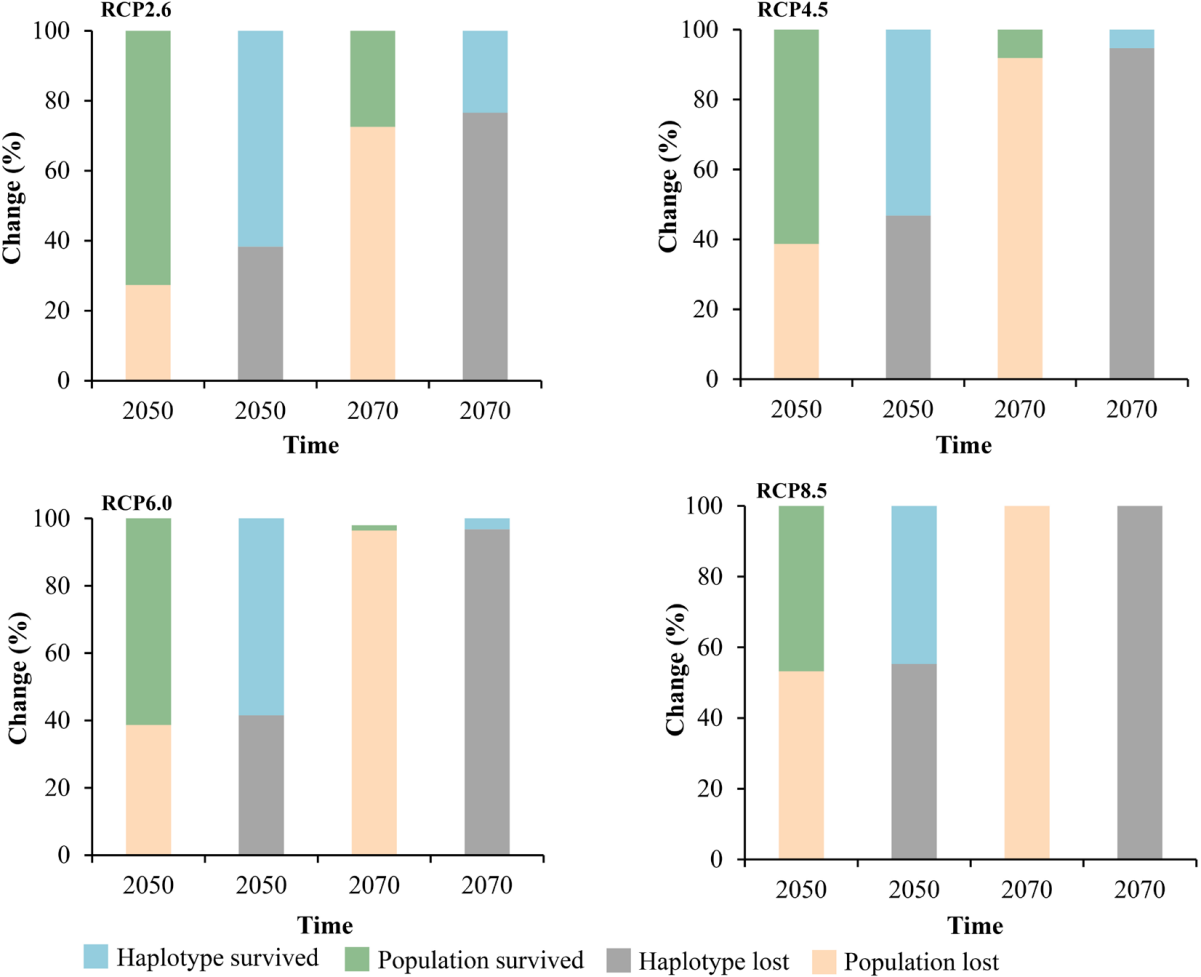
Percent loss of sampling localities and haplotypes of *O. kweichowensis* under four climate scenarios. “Survived” refers to sampling localities retaining suitable habitat and haplotypes persisting in those localities; “lost” refers to localities projected to lose suitability and associated haplotypes.

We have forecasted potential shifts in the suitable range size of *O. kweichowensis* under various climate scenarios (Figure 5). In 2050, the suitable habitat area for *O. kweichowensis* both decreased and increased. Notably, under the RCP2.6, the area of habitat expansion (47.1 × 103 km^2^) exceeded that of contraction (37.2 × 103 km^2^), suggesting that lower carbon dioxide emissions could lead to a net increase in the species' viable habitat (see Table 1). In contrast, in the other three scenarios, the area of expansion was less than that of contraction, highlighting the diversity of the species' habitat response to different climate futures. In 2070, except for the RCP4.5 scenario, the suitable habitat for *O. kweichowensis* is expected to expand in the remaining three scenarios. However, compared with 2050, the contraction of the suitable habitat is more pronounced under all four carbon dioxide emission scenarios, with the contraction area exceeding 150 × 10^3^ km^2^, indicating potential significant habitat loss for the species as the century progresses. Moreover, the direction of habitat contraction varies by scenario. Under the RCP2.6 and RCP4.5 scenarios, the suitable habitat is predicted to contract toward the central region of Guizhou, possibly related to the relatively lower impact of climate change under these emission pathways. Conversely, under the RCP6.0 and RCP8.5 scenarios, the habitat is forecasted to contract severely toward eastern Guizhou, with net losses of 172.6 × 10^3^ km^2^ (RCP6.0) and 179.4 × 10^3^ km^2^ (RCP8.5) by 2070 (Figure 6 and Table S6). This represents > 95% habitat reduction from current levels, responding to intensified climate stressors including prolonged droughts and extreme thermal events.

**TABLE 1:** Comparison of haplotype and nucleotide diversity change current to future.

Time	Scenarios	Nucleotide diversity	No. of haplotypes	Haplotype diversity
Current	Current	0.00082	94	0.674
2050	RCP2.6	0.00068	58	0.647
	RCP4.5	0.00069	50	0.668
	RCP6.0	0.00065	55	0.647
	RCP8.5	0.00075	42	0.694
2070	RCP2.6	0.00074	22	0.679
	RCP4.5	0.00047	5	0.554
	RCP6.0	0.00046	3	0.590
	RCP8.5	0.00000	0	0.000

### 3.6 Magnitude of Climate Change Impacts on the Diversity

We projected the genetic diversity of *O. kweichowensis* under future climate scenarios. In 2050, with the exception of the RCP8.5 scenario, the number of haplotypes saw a loss exceeding 50%; the other three scenarios demonstrated losses of less than 50%. In 2070, apart from the RCP2.6 scenario, the remaining three scenarios exhibited a substantial decrease in *O. kweichowensis* sampling localities and haplotypes. Under the RCP4.5 scenario, 9.1% of sampling localities and 5.3% of haplotypes are projected to persist; under the RCP6.0 scenario, 1.6% of sampling localities and 3.2% of haplotypes are projected to persist, and under the RCP8.5 scenario, *O. kweichowensis* is anticipated to lose all its populations and all haplotypes (Figures 5 and 6). Nucleotide diversity analysis shows that climate change has little impact on the nucleotide diversity of *O. kweichowensis* before 2050. Similarly, under the RCP2.6 scenario, in 2070, *O. kweichowensis* still possesses a high level of genetic diversity. However, with the increase in carbon dioxide emissions, the genetic diversity of *O. kweichowensis* is decreasing; under the RCP4.5 scenario, the nucleotide diversity has decreased to 0.00047, and under the RCP8.5 scenario, no populations or haplotypes are projected to persist (Table 1).

## 4 Discussion

Southwest China, a global biodiversity hotspot, harbors exceptional karst‐endemic biodiversity, yet the ecological drivers shaping its evolution remain understudied (Wen et al. 2022). This study focuses on *Odorrana kweichowensis* , a karst‐specialized frog, to unravel the interplay of geological processes and climate change in shaping its population dynamics, insights critical for conserving vulnerable karst ecosystems. By integrating phylogeography, ENMs, and genetic diversity projections, we reveal how climate fluctuations, rather than ancient geological events, have driven the species' recent expansion and highlight urgent conservation needs amid future climate change.

### 4.1 Post‐LGM Climate Warming as the Primary Expansion Driver

While Miocene orogenesis (e.g., Tibetan Plateau uplift) forged southwest China's karst topography (Zhang and Fang 2012; Ding et al. 2017), our results demonstrate that the demographic history of *O. kweichowensis* reflects younger, climate‐driven processes. Key evidence includes: (1) a star‐like haplotype network (Figure 2A) and lack of phylogenetic structure (Figure 2B), consistent with rapid expansion rather than vicariance; (2) the dominance of haplotype H1 (occurring at 55.6% frequency across 54 localities), indicating panmixia, with regional haplotypes (H2, H25) confined to northeastern Guizhou and the Guangxi–Guizhou corridor suggesting post‐LGM dispersal routes; (3) demographic signals (including significantly negative Tajima's D and Fu's FS values, a unimodal mismatch distribution, and a BSP‐inferred expansion ~15 kya; Figure 4) coinciding with post‐LGM warming; and (4) ENM reconstructions revealing an absence of suitable habitat during the LIG, refugial persistence within montane karst blocks (e.g., Wuling, Miaoling Mountains) during the LGM, and rapid post‐LGM expansion (Figure S5), mirroring patterns observed in other karst endemics like *Shinisaurus crocodilurus* (Zhang et al. 2022).

This pattern contrasts with that of ancient karst taxa (e.g., *Sinocyclocheilus* cavefishes), whose diversification aligns with geological events (Wen et al. 2022). Instead, *O. kweichowensis* exemplifies young radiations in which climate oscillations override older landscape effects (Rizvanovic et al. 2019), with karst heterogeneity facilitating, rather than dictating, its expansion. The wide range of pairwise Fst values (0.000–0.778) despite low overall genetic structure likely reflects stochastic divergence within isolated karst microhabitats, consistent with the nonsignificant isolation‐by‐distance pattern (Mantel test: *r* = 0.1302, *p* = 0.101).

### 4.2 Key Environmental Constraints and Climate Change Vulnerability

ENMs identified three critical factors limiting *O. kweichowensis* distribution: the mean temperature of the driest quarter (Bio9, 33.8% contribution), precipitation of the driest quarter (Bio17, 26.7% contribution), and altitude (15.8% contribution). The species' narrow adaptation ranges, Bio9: −2.5°C to 6.6°C, Bio17: 74.1 to 148.1 mm, and altitude ≤ 1040 m (Figure S4), reflect its dependence on karst microclimates that buffer dry‐season extremes. These constraints are ecologically meaningful: Bio9 and Bio17 directly regulate karst aquifer recharge and stream persistence, while low altitudes ensure mild temperatures critical for larval development (Gao et al. 2015).

Future projections under climate scenarios highlight stark risks. By 2050, only RCP2.6 (low emissions) shows net habitat expansion (47.1 × 10^3^ km^2^ vs. contraction of 37.2 × 10^3^ km^2^; Table S6), whereas higher‐emission scenarios (RCP4.5–8.5) predict net loss. By 2070, RCP8.5 characterized by extreme warming leads to > 95% habitat loss (179.4 × 10^3^ km^2^ contraction), driven by Bio9 exceeding 6.6°C and Bio17 dropping below 74.1 mm (Figure 5). This aligns with broader evidence that amphibians, with permeable skin and temperature‐dependent development, are hypersensitive to CO_2_‐driven climate shifts (Carey and Alexander 2003; Li et al. 2013).

### 4.3 Genetic Diversity at Risk: Implications for Resilience

Genetic diversity, a cornerstone of adaptive potential (Hu et al. 2021), is projected to decline sharply with rising emissions. By 2050, all scenarios except RCP8.5 retain > 50% of haplotypes, but by 2070, only RCP2.6 preserves 22 haplotypes and high nucleotide diversity (0.00074; Table 1). In contrast, RCP8.5 predicts complete loss of haplotypes and genetic diversity. The southwest Guizhou region with the highest haplotype (Hd = 1.00) and nucleotide (*π* = 0.00319) diversity (Figure 3) emerges as a critical genetic hotspot, yet it is not fully protected, underscoring the need for targeted conservation.

### 4.4 Conservation Implications and Policy Alignment

China's Wildlife Protection Law (2023) and Biodiversity Conservation Strategy (2021–2030) prioritize species and ecosystem protection but lack explicit provisions for intraspecific genetic diversity. Our results call for integrating genetic data into policy: expand reserves to include southwest Guizhou and the Guangxi–Guizhou border (H25 haplotype cluster), where diversity is highest; designate central Guizhou, projected to retain suitable habitat under RCP2.6 (Figure 4) as a climate‐resilient core area; and establish corridors between fragmented sampling localities (e.g., the KL contact zone) to maintain gene flow, countering the absence of significant isolation by distance (Mantel test *R* = 0.1302, *p* = 0.101).

### 4.5 Limitations and Future Directions

While mitochondrial data revealed broad phylogeographic patterns, the maternal inheritance of mtDNA limits insights into male‐mediated gene flow or nuclear‐based adaptive processes, potentially underestimating the complexity of *O. kweichowensis* ' evolutionary dynamics. To address these gaps, future studies should first apply genome‐wide single‐nucleotide polymorphisms (SNPs), leveraging the buccal swab sampling method to detect cryptic population structure and identify genes associated with drought tolerance or thermal adaptation, traits critical for survival in karst microhabitats. Second, integrating genetic offset models with ENMs will help pinpoint populations at the highest risk of adaptive mismatches under future climate scenarios, refining conservation priorities for this karst endemic species.

## Author Contributions

**Li Shize:** data curation (equal), formal analysis (equal), investigation (equal), methodology (equal), software (equal), writing – original draft (lead). **Liu Jing:** data curation (equal). **Su Haijun:** funding acquisition (equal), writing – review and editing (equal). **Wei Gang:** project administration (equal), writing – review and editing (equal). **Mu Lang:** data curation (equal). **Shen Tuo:** data curation (equal). **Xu Houqiang:** project administration (equal), writing – review and editing (lead).

## Conflicts of Interest

The authors declare no conflicts of interest.

## Supporting information


**Figure S1:** Pearson correlation matrix of 19 environmental variables and elevation.

**Figure S2:** Mantel test of geographical distance and genetic distance.

**Figure S3:** AUC value obtained from ROC analysis to test model predictions.

**Figure S4:** Response curve of environmental variables to model prediction.

**Figure S5:** Potential distribution of *O. kweichowensis* under current and past (A) LIG. (B) LGM. (C) MID. (D) CUR.

**Table S1:** Information for samples used in molecular phylogenetic analyses in this study.

**Table S2:** Occurrence from field survey and literatures used for ENMs analysis in this study.

**Table S3:** Bioclimatic variables for ENMs.

**Table S4:** Population distribution, codes, haplotypes, and genetic diversity of *O. kweichowensis* .

**Table S5:** F‐statistics (Fst) among the sampling localities of *O. kweichowensis* .

**Table S6:** Suitable range size changes under the future climate scenarios of *O. kweichowensis* (10^3^ km^2^).

## Data Availability

All the sequencing data was uploaded to the National Center for Biotechnology Information (NCBI); the accession numbers are PP812697–PP813412 for COI and PP813876–PP814591 for ND2, and the R code used in this study was uploaded to figshare (https://figshare.com/s/9b0565430e094c02c6ef).
