# Supplementary figures and images for: Phylogeography and Species Distribution Modeling of Guizhou Odorous Frog ( Odorrana kweichowensis ) Endemic to Southern China Karst Region
Source: Ecol Evol. 2025 Sep 14;15(9):e72160. doi: 10.1002/ece3.72160 (PMC12434189; doi:10.1002/ece3.72160)

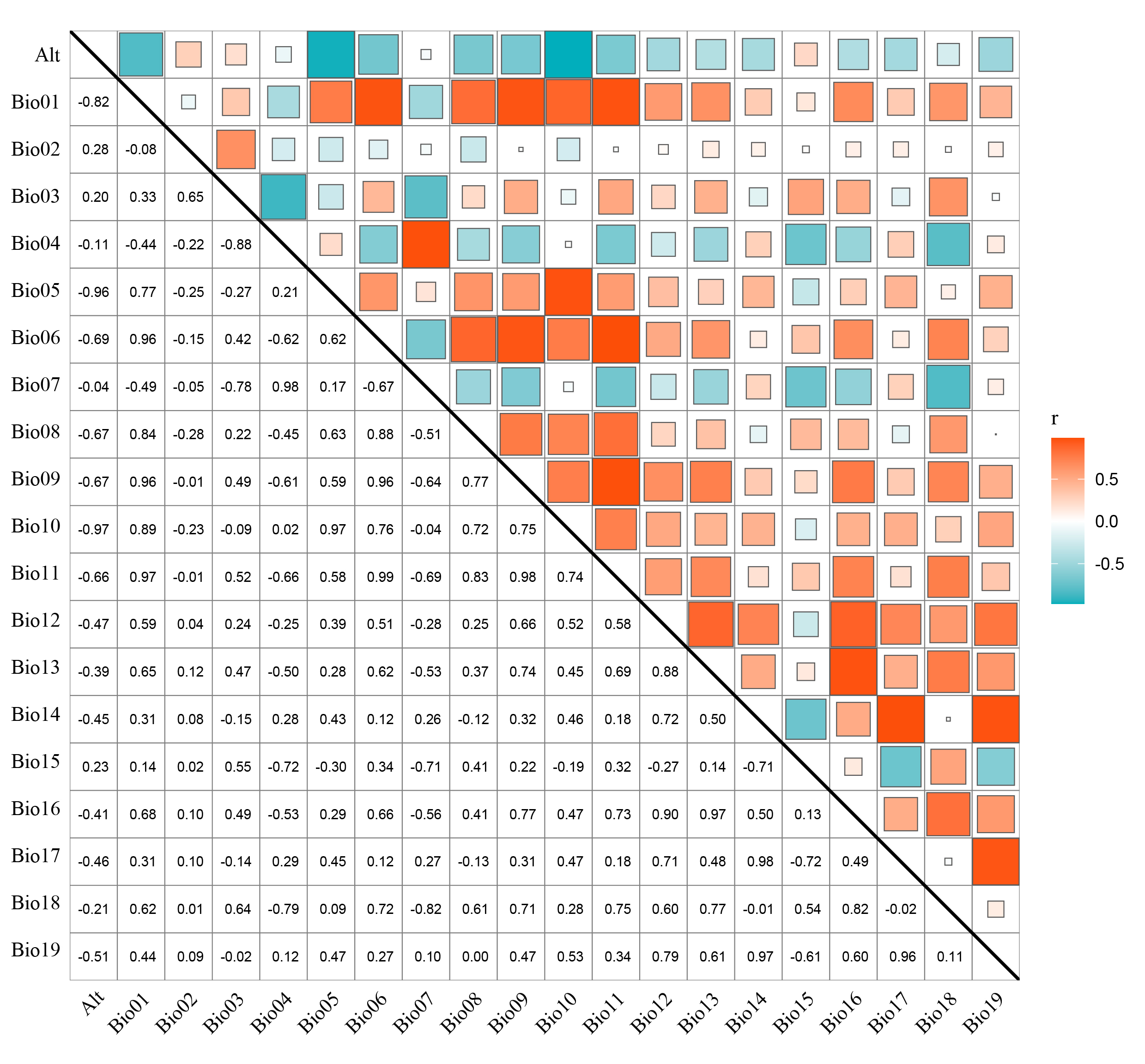

Supplement: Supplementary file 1 — Figure S1: Pearson correlation matrix of 19 environmental variables and elevation. [file ECE3-15-e72160-s008.jpg]

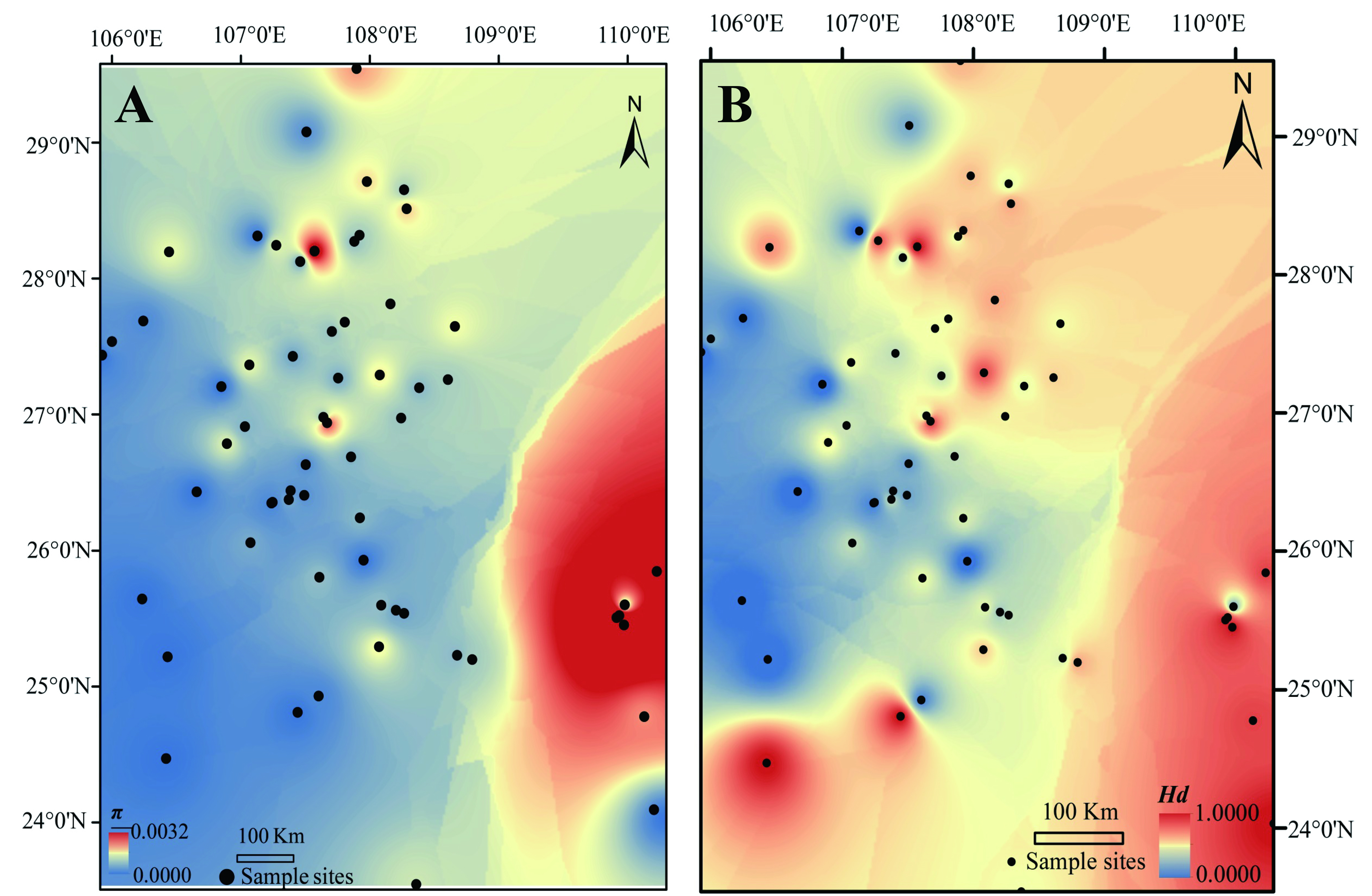

Supplement: Supplementary file 2 — Figure S2: Mantel test of geographical distance and genetic distance. [file ECE3-15-e72160-s010.jpg]

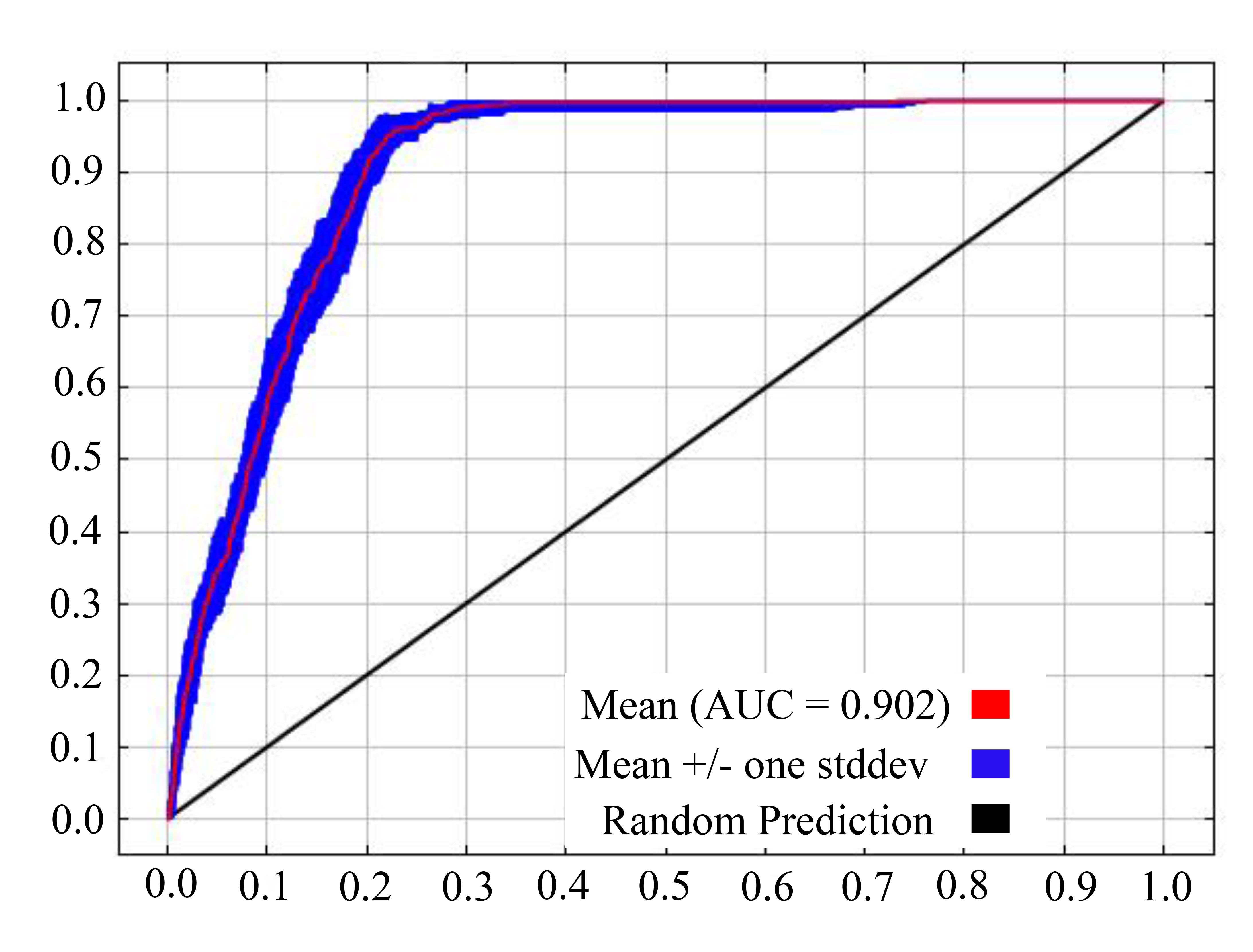

Supplement: Supplementary file 3 — Figure S3: AUC value obtained from ROC analysis to test model predictions. [file ECE3-15-e72160-s002.jpg]

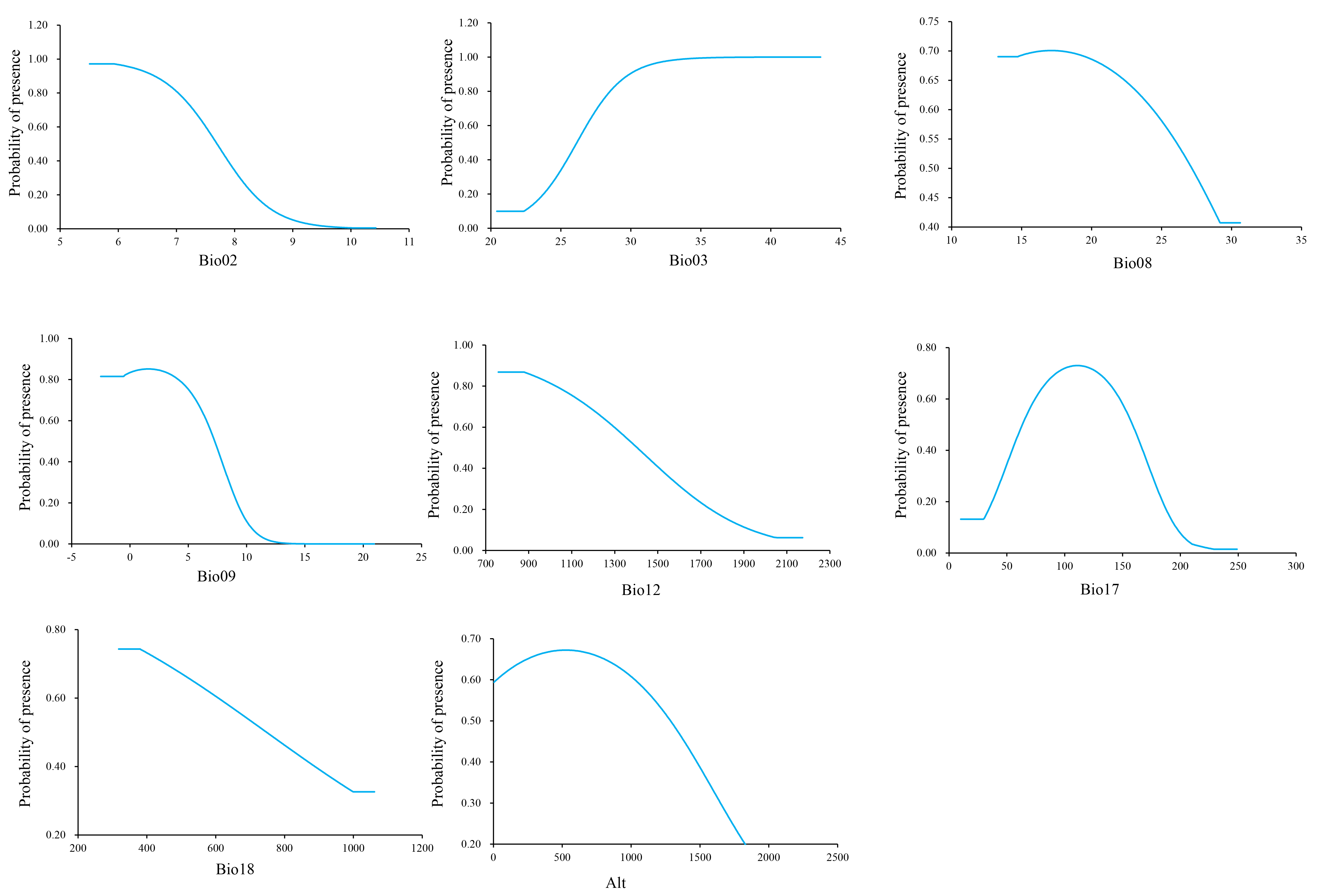

Supplement: Supplementary file 4 — Figure S4: Response curve of environmental variables to model prediction. [file ECE3-15-e72160-s011.jpg]

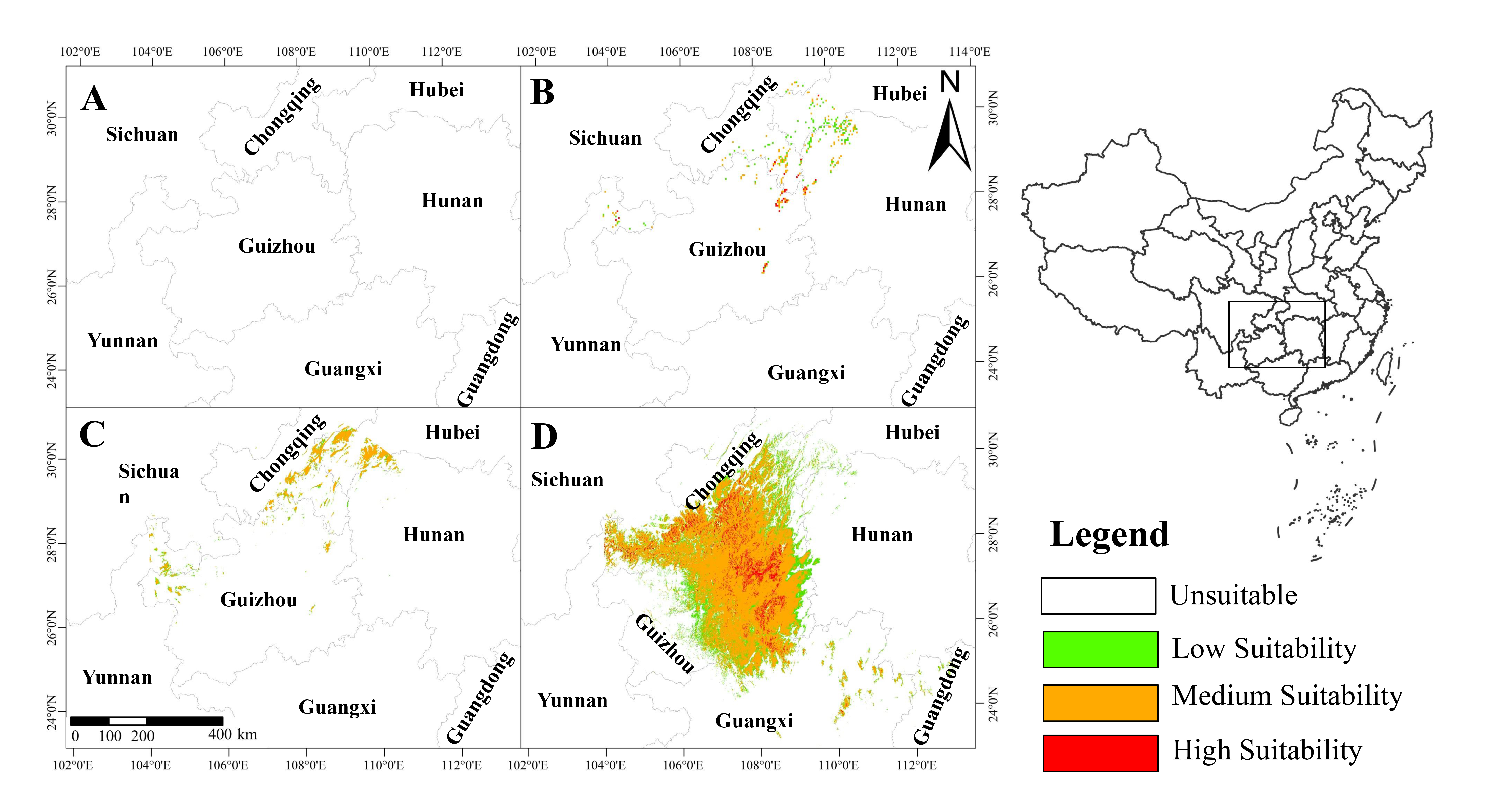

Supplement: Supplementary file 5 — Figure S5: Potential distribution of O. kweichowensis under current and past (A) LIG. (B) LGM. (C) MID. (D) CUR. [file ECE3-15-e72160-s001.jpg]
